# A plant-based meal reduces postprandial oxidative and dicarbonyl stress in men with diabetes or obesity compared with an energy- and macronutrient-matched conventional meal in a randomized crossover study

**DOI:** 10.1186/s12986-021-00609-5

**Published:** 2021-09-10

**Authors:** Hana Malinska, Marta Klementová, Michaela Kudlackova, Jiri Veleba, Eva Hoskova, Olena Oliyarnyk, Irena Markova, Lenka Thieme, Martin Hill, Terezie Pelikanova, Hana Kahleova

**Affiliations:** 1 Institute for Clinical and Experimental Medicine, Prague, Czech Republic; 2 Institute of Endocrinology, Prague, Czech Republic; 3 Physicians Committee for Responsible Medicine, 5100 Wisconsin Ave, NW, Suite 400, Washington, DC 20016 USA

**Keywords:** Postprandial state, Type 2 diabetes, Plant-based diet, Inflammation, Methylglyoxal, Oxidative stress, Appetite hormones

## Abstract

**Background:**

Increased oxidative/dicarbonyl stress and chronic inflammation are considered key pathophysiological mediators in the progression of complications in obesity and type 2 diabetes (T2D). Lifestyle and diet composition have a major impact. In this study, we tested the effects of a vegan (V) and a conventional meat containg (M) meal, matched for energy and macronutrients, on postprandial oxidative and dicarbonyl stress, inflammatory markers and appetite hormones.

**Methods:**

A randomised crossover design was used to evaluate T2D, obese with normal glucose tolerance and control participants (n = 20 in each group), with serum concentrations of analytes determined at 0, 120 and 180 min. Repeated-measures ANOVA was used for statistical analysis.

**Results:**

In T2D subjects, we observed decreased postprandial concentrations of oxidised glutathione (*p *˂ 0.001) and increased glutathione peroxidase activity (*p* = 0.045) after the V-meal consumption, compared with the M-meal. In obese participants, V-meal consumption increased postprandial concentrations of reduced glutathione (*p* = 0.041) and decreased methylglyoxal concentrations (*p* = 0.023). There were no differences in postprandial secretion of TNFα, MCP-1 or ghrelin in T2D or obese men, but we did observe higher postprandial secretion of leptin after the V-meal in T2D men (*p* = 0.002) compared with the M-meal.

**Conclusions:**

The results show that a plant-based meal is efficient in ameliorating the postprandial oxidative and dicarbonyl stress compared to a conventional energy- and macronutrient-matched meal, indicating the therapeutic potential of plant-based nutrition in improving the progression of complications in T2D and obese patients.

Registered under ClinicalTrials.gov Identifier No. NCT02474147.

**Supplementary Information:**

The online version contains supplementary material available at 10.1186/s12986-021-00609-5.

## Introduction

Type 2 diabetes (T2D) and obesity are associated with postprandial dysmetabolism, a state that significantly contributes to the development of associated complications [1]. It has been recently suggested that non-fasting glucose and, particularly, postprandial concentrations of blood glucose and lipids are reliable predictors of cardiovascular disease [2, 3]. A prolonged postprandial period may induce pro-oxidant conditions and pro-inflammatory activity, both of which are implicated in micro- and macrovascular damage [1].

Abnormal postprandial elevations of plasma glucose and lipids in T2D are associated with oxidative stress, increased inflammation, hypercoagulation and impaired secretion of gastrointestinal hormones [4, 5]. Our previous findings from a study involving T2D patients showed that postprandial oxidative stress occurred independently of alterations in gastrointestinal peptides [6]. It has been recently suggested that postprandial formation of dicarbonyl compounds, toxic reactive metabolites from glucose and lipids, may be involved in the development of vascular complications of diabetes [7]. Dicarbonyls interact with proteins to form advanced glycation end products, which can damage the endothelium and impair vasorelaxation [8].

The management of postprandial dysmetabolism represents an important strategy in the treatment of T2D and its complications. One important element of this approach is diet composition. The preventative and therapeutic effects of vegetarian diets on diabetes have been examined over recent decades and have been shown at least as beneficial as pharmacotherapy in diabetes management [9]. Vegetarian diets are inversely associated with the risk of developing diabetes and are associated with two-fold lower the prevalence of T2D [10]. In a previous clinical interventional study, a vegetarian diet led to greater weight loss and improved glycemic control, insulin resistance and oxidative stress markers compared to a conventional hypocaloric diet [11]. Furthermore, our previous randomised crossover study reported that a plant-based meal increased postprandial secretion of gastrointestinal hormones more effectively than a proceseed meat meal in obese and T2D men [12]. Another study found improvements in postprandial incretin and insulin secretion after a plant-based meal in T2D patients, highlighting the therapeutic potential of this type of diet in improving β-cell function [13]. In this secondary analysis of previously published data [14], we have tested postprandial response to a plant-based meal compared with an energy- and macronutrient-matched conventional meal on oxidative and dicarbonyl stress, inflammation and appetite hormones in T2D and obese patients. Our hypothesis was that a plant-based meal would stimulate postprandial appetite hormones secretion and produce lower postprandial response of oxidative and dicarbonyl stress compared with a conventional meal, particularly by decreasing the serum concentrations of oxidized glutathione (primary outcome), and by decreasing glutathione peroxidase activity and reduced glutathione (secondary outcomes). The results of the study may help build the evidence base for dietary guidelines for T2D and obese patients.

## Materials and methods

### Study subjects and design

The methods have beedn described in detail previously [14]. Breinfly: This randomised crossover study group included 20 men with T2D, 20 obese men with normal glucose tolerance, and 20 healthy men. All individuals consumed two energy- and macronutrient-matched test meals in random order. The general metabolic characteristics of each group are given in Table 1. Written informed consent was obtained from all participants prior to enrolment in the study. The study was approved by the ethics committees of Thomayer Hospital and the Institute for Clinical and Experimental Medicine, Prague, Czech Republic (protocol identification number G14-08-42). The study was prospectively registed at ClinicalTrials.gov (Identifier: NCT02474147).

**Table 1:** General characteristics of the study population

	T2D	Obese	Controls
	n = 20	n = 20	n = 20
Age (years)	47.8 ± 8.2	43.0 ± 7.0	42.7 ± 7.1^#^
Weight (kg)	108.2 ± 11.9	103.4 ± 13.3^¶¶¶^	77.4 ± 8.1^###^
Body mass index (kg/m^2^)	34.5 ± 11.9	32.7 ± 3.9^¶¶¶^	23.8 ± 1.5^###^
Waist circumference (cm)	106.9 ± 23.6	109.0 ± 8.5^¶¶¶^	85.0 ± 5.3^###^
HbA1c (mmol/mol)	48.5 ± 8.1***	36.4 ± 3.0	36.1 ± 3.2^###^
Fasting plasma glucose (mmol/l)	7.2 ± 1.5***	5.1 ± 0.3	5.1 ± 0.4^###^
Triglycerides (mmol/l)	2.1 ± 1.1	2.2 ± 1.1^¶¶¶^	1.1 ± 0.6^##^
LDL-cholesterol (mmol/l)	2.6 ± 0.1*	3.3 ± 0.7	2.8 ± 0.7
HDL-cholesterol (mmol/l)	1.0 ± 0.2	1.0 ± 0.3^¶¶¶^	1.5 ± 0.2^###^
Systolic blood pressure (mm Hg)	144.4 ± 13.4*	134.8 ± 7.6^¶¶^	124.0 ± 11.4^###^
Diastolic blood pressure (mm Hg)	96.2 ± 8.8*	90.0 ± 6.8^¶¶¶^	80.7 ± 5.6^###^
Duration of diabetes (years)	4.3 ± 3.3	–	–

Adapted and published in our previous part of the study [13]

Significant difference between the groups of participants: * is used for difference between T2D and obese participants (* denotes *p*˂0.05, *** denotes *p*˂0.001), ¶ is used for difference between obese and control men (¶¶ denotes *p*˂0.01, ¶¶¶ denotes *p*˂0.001), # is used for difference between T2D and control men (## denotes *p*˂0.01, ### denotes *p*˂0.001)

All participants were male and of Czech nationality. Men with T2D and at least three hallmarks of metabolic syndrome (30–65 years of age; BMI, 25–45 kg/m^2^; HbA1c, 42–105 mmol/mol) were treated by lifestyle alone or with oral hypoglycemic agents (metformin and/or sulfonylureas) for at least 1 year. The obese men were BMI- and age-matched to men with T2D; the healthy men comprised age-matched controls with normal BMI (between 19 and 25 kg/m^2^) and normal glucose tolerance. Exclusion criteria were thyroid, liver or kidney disease, drug or alcohol abuse, unstable drug therapy, or significant weight loss of more than 5% body weight in the preceding three months.

### Randomization and interventions

The participants were randomly assigned in a 1:1 ratio a vegan meal or an energy- and marconutrient-matched conventional meat containing meal based on a computer-generated randomisation protocol. The randomization protocol was designed so as not to be accessed beforehand, with the interventions unmasked. Outcome assessors were blinded to the interventions.

All participants fasted for 10–12 h overnight. Diabetic patients avoided diabetes medication the evening before, and on the morning of, the assessment. The meal consisted of either a conventional meat burger (M-meal) or a vegan burger (V-meal). Both meals were consumed with a hot caffeinated beverage. The composition of the test meals is given in Table 2. Each time, the participants were asked to eat the whole test meal. Tap water was allowed ad libitum. Unaware of the sequence of interventions, participants arrived at the laboratory in the morning to be assigned one of the randomized test meals. The participants were checking in for their meal assessments between 7–8:30 am. They usually finished their meal in 15–20 min. The whole meal test took 3 h from finishing their meal. After a washout period of one week of staying on their usual meal plan, the participants returned to complete the opposite test meal. The participants were instructed not to change their usual dietary habits or physical activity during the study. All participants found both meals acceptable and nobody complained about any particular meal.

**Table 2:** Composition of the test meals

	V-meal	M-meal
Total weight (g)	200	200
Energy content (kJ/kcal)	2154 (514.9)	2149 (513.6)
Carbohydrates (g) (%)	54.2 (44%)	55 (44.8%)
Proteins (g) (%)	19.9 (16.2%)	20.5 (16.7%)
Total fat (g) (%)	22.8 (39.8)	22 (38.6%)
Saturated fatty acids (g)	2.2	8.6
Fibre (g)	7.8	2.2

The postprandial state was measured after intake of a standard breakfast—one of two energy- and macronutrient-matched meals in a random order: either a plant-based meal (V-meal; tofu burger with spices, ketchup, mustard, tomato, lettuce and cucumber in a wheat bun) together with 300 mL of green tea, or a conventional meat meal (M-meal; cooked-pork seasoned meat in a wheat bun, tomato, cheddar-type cheese, lettuce, spicy sauce) together with 300 mL Café Latte [14]

### Analytical methods

Anthropometric measurements and blood pressure: Height and weight were measured using a stadiometer with a calibrated scale accurate to 0.1 kg. Ensuring participants had been in a seated position be forehand, blood pressure was measured using the M6 Comfort digital monitor on three occasions at 2-min intervals. A mean value was calculated from the last two measurements. Blood samples were drawn in the fasting state and then 30, 60, 120 and 180 min after the standard meal. After that, the samples were centrifuged and aliquots of plasma/serum were stored at − 80 °C for analysis. Plasma glucose was analysed using the Beckman Analyzer glucose-oxidase method (Beckman Instruments, Inc., Fullerton, CA, USA) and glycated haemoglobin using the VARIANT II Hemoglobin Testing System (Bio-Rad Laboratories GmbH, Munich, Germany). Plasma lipids were measured using enzymatic methods (Roche, Basel, Switzerland).

Inflammatory markers and appetite hormones: Concentrations of TNFα, MCP-1, leptin and ghrelin were determined by multiplex immunoanalysis based on xMAP technology using the MILLIPLEX MAP Human Metabolic Hormone Magnetic Bead Panel (HMHEMAG-34 K) (Millipore, Billerica, MA, USA) and the Luminex 100 IS instrument (Luminex Corporation, Austin, USA).

Oxidative stress markers: The whole blood level of reduced and oxidised forms of glutathione (oxidized from is the primary outcome and reduced form a secondary outcome) were determined using the Glutathione in Whole Blood—HPLC diagnostic kit (Chromsystems, Munich, Germany). The activity of glutathione peroxidase (GPx, secondary outcome) was analysed using a glutathione peroxidase assay kit (Cayman Chemical, MI, USA). The serum level of ascorbic acid was measured using a spectrophotometric method as previously described [6].

Dicarbonyl stress markers: The concentration of methylglyoxal was determined after derivatisation with 1,2-diaminobenzene using HPLC and fluorescence detection according to Fleming and Bierhaus as previously described [15]. A registered dietitian analyzed both meals, using a country-speficif food database and software [16].

### Statistical analysis

Sample size was estimated based on a power analysis with an alpha of 0.05 and a power of 0.80 to detect between intervention differences in serum concentrations of oxidized glutathione (primary outcome), using the PASS 16.0 Power analysis and sample size software, 2018 (NCSS, Kaysville, UT, USA). Based on our preliminary data, after data transformation to achieve normal distribution, to have 80% power to detect a difference between the two meals would require 10 subjects in each group for the primary outcome and 14 subjects in each group were required for the secondary outcomes (glutathione peroxidase activity and reduced glutathione). Assuming an attrition of 25%, the expected sample size is 18 in each group. Having 20 participants in each group would give us 93% power.

Intention to treat analysis was performed, using repeated-measures ANOVA. Group, subject and time factors were all included in the model as follows: inter-individual (T2D vs. obese vs. controls); intra-individual (time taken to complete the meal test); and interaction between factors (divergence degree between the time profiles of each group). Sequence of meals was tested in a separate model and was not significant in any of the tested outcomes.

To eliminate skewed data distribution and heteroscedasticity, the original data was transformed to a Gaussian distribution to attain symmetric distribution in both predictors and dependent variables and, at the same time, to stabilize the variance (attaining homoscedasticity), by a power transformation using the statistical software Statgraphics Centurion, version XV from Statpoint Inc. (Herndon, Virginia, USA), as descibed in detail previously [17]. After performing the statistical tests, the data were then retransformed into the original scale, using a recurrent formula. For posthoc analysis, Duncan test for multiple comparisons with the correction for multiplicity was used. Analysis was carried out using PASS 2005 statistical software (Number Cruncher Statistical Systems, USA); the statistician was blinded to the meal assignement. Data are presented as the mean with 95% confidence intervals (CI).

## Results

The flow-chart showing the recruitment and follow-up of the participants is in Additional file 1: Figure S1. The general characteristics for the individual groups of participants are shown in Table 1, with the macronutrient content of the test meals shown in Table 2. The meals were eaten in full by all study participants.

**Fig. 1:**
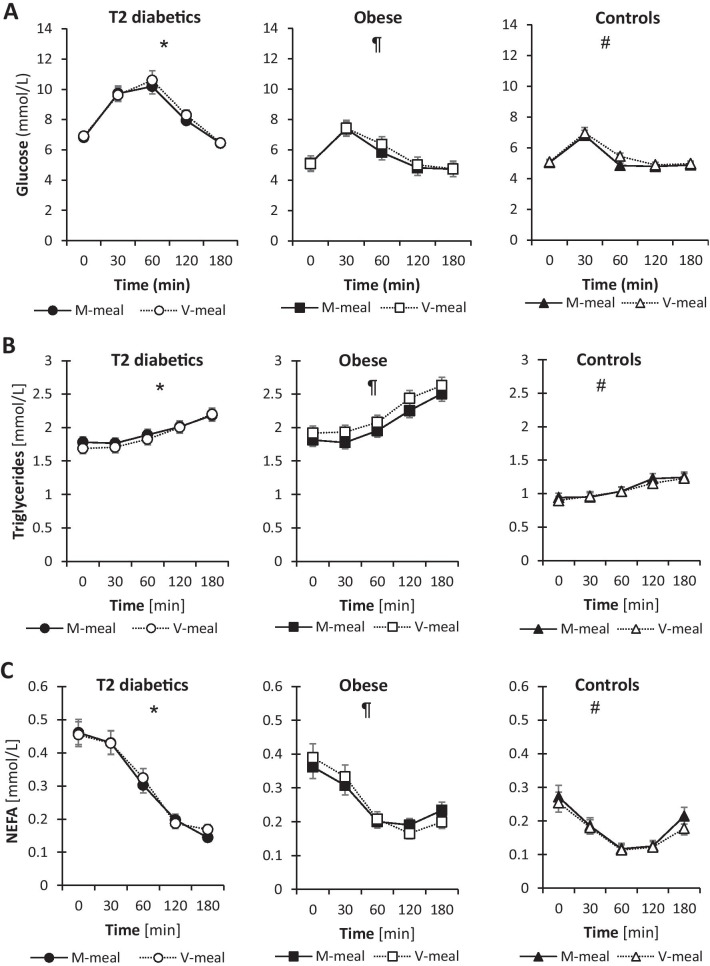
Postprandial changes in plasma concentrations of glucose and lipids in T2D, obese and control subjects after a standard meal test consisting of either a conventional meat burger (M-meal) or a plant-based tofu burger (V-meal). Data are expressed as the mean with a 95% CI. * indicates the difference between T2D and obese men, ¶ difference between obese and control men, and # difference between T2D and control men at α = 0.05. (**A**) Plasma glucose (*, ¶, #). T2D: Meal: F = 1, *p* = 0.309; Time: F = 118.1, *p* < 0.001; Meal × Time: F = 0.3, *p* = 0.889. Obese: Meal: F = 3.6, *p* = 0.06; Time: F = 151.8, *p* < 0.001; Meal × Time: F = 1.5, *p* = 0.192. Controls: Meal: F = 6.2, *p* = 0.014; Time: F = 59.7, *p* < 0.001; Meal × Time: F = 1.7, *p* = 0.143. (**B**) Plasma triglycerides (*, ¶, #). T2D: Meal: F = 1.3, *p* = 0.259; Time: F = 20.9, *p* < 0.001; Meal × Time: F = 0.3, *p* = 0.887. Obese: Meal: F = 8.4, *p* = 0.004; Time: F = 34, *p* < 0.001; Meal × Time: F = 0.1, *p* = 0.987. Controls: Meal: F = 0.6, *p* = 0.432; Time: F = 16.4, *p* < 0.001; Meal × Time: F = 0.2, *p* = 0.921. (**C**) Non-esterified fatty acids (NEFA) (*, ¶, #). T2D: Meal: F = 0.6, *p* = 0.432; Time: F = 126.3, *p* < 0.001; Meal × Time: F = 0.9, *p* = 0.469. Obese: Meal: F = 0.2, *p* = 0.632; Time: F = 40.9, *p* < 0.001; Meal × Time: F = 1.4, *p* = 0.239. Controls: Meal: F = 1.5, *p* = 0.223; Time: F = 31.1, *p* < 0.001; Meal × Time: F = 0.3, *p* = 0.848

As shown in Fig. 1, there were no differences in plasma concentrations of glucose, triglycerides or free fatty acids between the M-meal and V-meal across all groups during the postprandial state, except for plasma glucose being slightly higher at 60 min after the V-meal compared with the M-meal in healthy controls (but the values still being much lower compared with T2D and obese men), and triglycerides being higher at 120 min after the V-meal in obese men. In T2D men, we observed increased basal fasting levels in conjunction with higher changes and dynamics in the postprandial state for these parameters irrespective of the test meal. Plasma concentrations of triglycerides were inversely related to concentrations of free fatty acids.

### Oxidative and dicarbonyl stress parameters

**Fig. 2:**
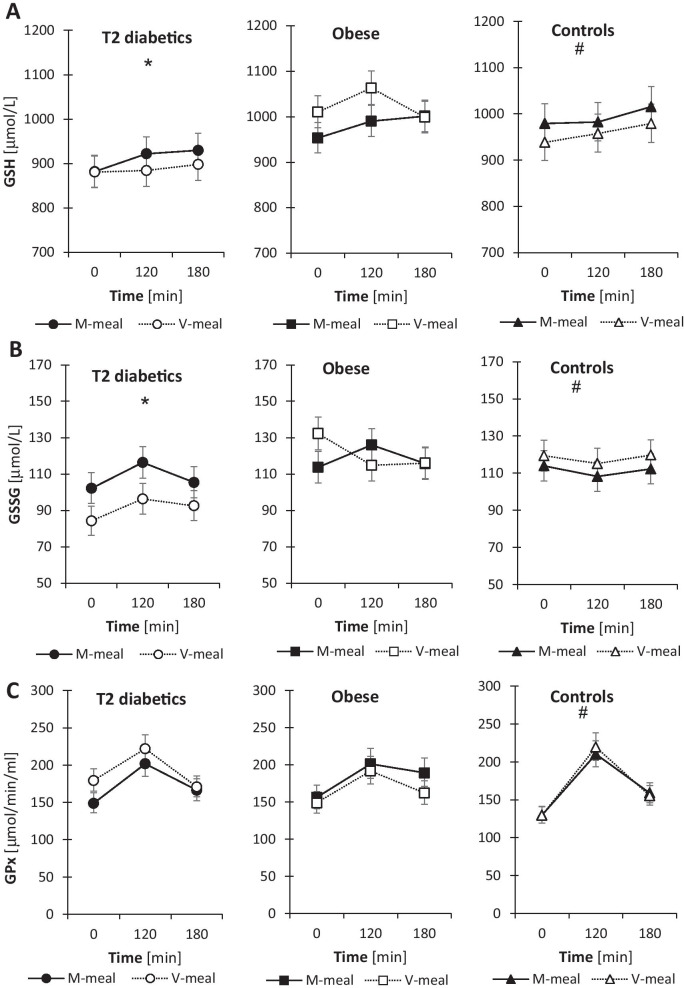

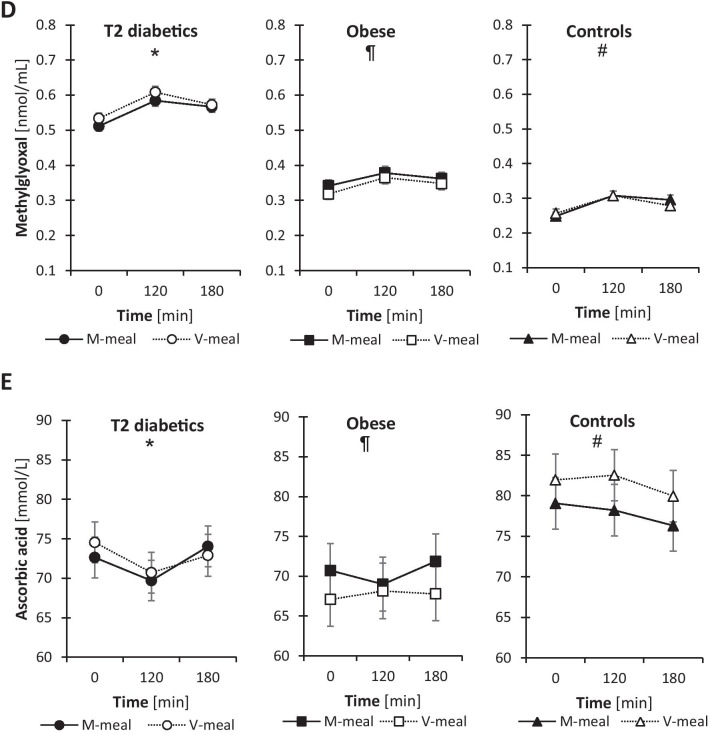
Postprandial changes in plasma parameters of oxidative and dicarbonyl stress in T2D, obese and control subjects after a standard meal test consisting of either a conventional meat burger (M-meal) or a plant-based tofu burger (V-meal). Data are expressed as the mean with a 95% CI. *Indicates the difference between T2D and obese men, ¶ difference between obese and control men, and # difference between T2D and control men at α = 0.05. (**A**) Plasma GSH (*, #) T2D: Meal: F = 1.2, *p* = 0.274; Time: F = 0.8, *p* = 0.445; Meal × Time: F = 0.3, *p* = 0.757. Obese: Meal: F = 4.3, *p* = 0.041; Time: F = 1.6, *p* = 0.203; Meal × Time: F = 1.3, *p* = 0.293. Controls: Meal: F = 2, *p* = 0.161; Time: F = 0.9, *p* = 0.415; Meal × Time: F = 0, *p* = 0.961. (**B**) GSSG (*, #) T2D: Meal: F = 12, *p* < 0.001; Time: F = 2.4, *p* = 0.095; Meal × Time: F = 0.2, *p* = 0.835. Obese: Meal: F = 0.3, *p* = 0.62; Time: F = 0.7, *p* = 0.526; Meal × Time: F = 2.9, *p* = 0.062. Controls: Meal: F = 2, *p* = 0.166; Time: F = 0.4, *p* = 0.654; Meal × Time: F = 0, *p* = 0.984. (**C**) GPx activity (#). T2D: Meal: F = 4.1, *p* = 0.045; Time: F = 11, *p* < 0.001; Meal × Time: F = 0.9, *p* = 0.423. Obese: Meal: F = 2.1, *p* = 0.156; Time: F = 6.5, *p* = 0.002; Meal × Time: F = 0.4, *p* = 0.681. Controls: Meal: F = 0, *p* = 0.901; Time: F = 37, *p* < 0.001; Meal × Time: F = 0.2, *p* = 0.844. (**D**) Methylglyoxal (*, ¶, #). T2D: Meal: F = 3.7, *p* = 0.058; Time: F = 23, *p* < 0.001; Meal × Time: F = 0.4, *p* = 0.668. Obese: Meal: F = 5.4, *p* = 0.023; Time: F = 10.9, *p* < 0.001; Meal × Time: F = 0.3, *p* = 0.769. Controls: Meal: F = 0.1, *p* = 0.716; Time: F = 20, *p* < 0.001; Meal × Time: F = 1.1, *p* = 0.352. (**E**) Ascorbic acid (*, ¶, #). T2D: Meal: F = 0.2, *p* = 0.693; Time: F = 2.2, *p* = 0.122; Meal × Time: F = 0.4, *p* = 0.705. Obese: Meal: F = 2.1, *p* = 0.155; Time: F = 0.1, *p* = 0.869; Meal × Time: F = 0.3, *p* = 0.776. Controls: Meal: F = 3.9, *p* = 0.053; Time: F = 0.7, *p* = 0.502; Meal × Time: F = 0.1, *p* = 0.952

In general, after the plant-based V-meal, we observed improvements in oxidative and dicarbonyl stress parameters in the postprandial state, particularly in T2D patients (Fig. 2). We observed significantly decreased levels of the oxidised form of glutathione (GSSG; (*p* ˂ 0.001) and increased GPx activity (*p* = 0.045) in T2D men after the V-meal compared with the M-meal. In obese men, we observed an increase in postrandial levels of reduced glutathione (*p* = 0.041) and lower postprandial concentrations of methylglyoxal (*p* = 0.023) after the V-meal compared to M-meal. In T2D patients, there were no significant differences in methylglyoxal levels between the test meals, but postprandial methylglyoxal levels were markedly higher in men with T2D compared with obese and control subjects. In controls, postprandial levels of ascorbic acid tended to be more elevated after the V-meal compared to the M-meal (*p* = 0.053). The post-meal levels of ascorbic acid in T2D and obese participants were lower than in healthy controls irrespective of the test meal.

### Inflammatory parameters and appetite hormones

**Fig. 3:**
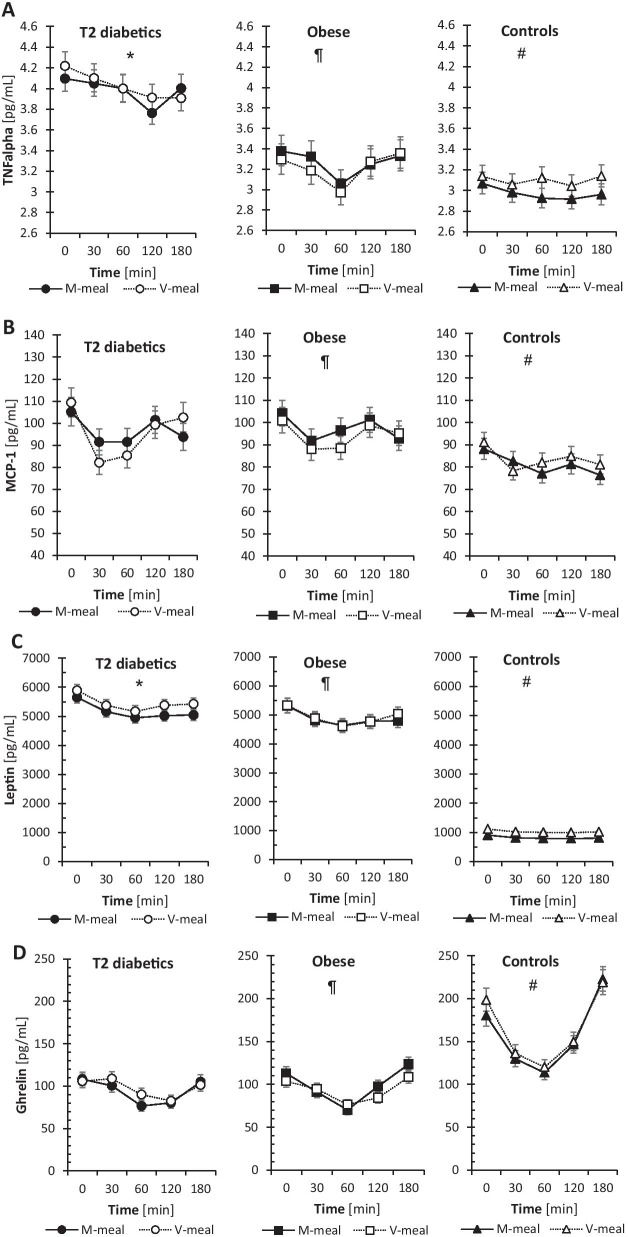
Postprandial changes in plasma inflammatory markers and appetite hormones in T2D, obese and control subjects after a standard meal test consisting of either a conventional meat burger (M-meal) or a plant-based tofu burger (V-meal). Data are expressed as the mean with a 95% CI. *Indicates the difference between T2D and obese men, ¶ difference between obese and control men, and # difference between T2D and control men at α = 0.05. (**A**) TNFα (*, ¶, #). T2D: Meal: F = 0.6, *p* = 0.44; Time: F = 3.7, *p* = 0.007; Meal × Time: F = 0.6, *p* = 0.664. Obese: Meal: F = 0.6, *p* = 0.434; Time: F = 3.6, *p* = 0.008; Meal × Time: F = 0.3, *p* = 0.91. Controls: Meal: F = 8.1, *p* = 0.005; Time: F = 0.8, *p* = 0.544; Meal × Time: F = 0.3, *p* = 0.864. (**B**) MCP-1 (¶, #). T2D: Meal: F = 0.2, *p* = 0.671; Time: F = 7.8, *p* < 0.001; Meal × Time: F = 1.5, *p* = 0.211. Obese: Meal: F = 1.5, *p* = 0.228; Time: F = 3.6, *p* = 0.008; Meal × Time: F = 0.5, *p* = 0.775. Controls: Meal: F = 1.5, *p* = 0.23; Time: F = 3.9, *p* = 0.005; Meal × Time: F = 0.8, *p* = 0.517. (**C**) Leptin (*, ¶, #). T2D: Meal: F = 10.2, *p* = 0.002; Time: F = 7.4, *p* < 0.001; Meal × Time: F = 0.2, *p* = 0.951. Obese: Meal: F = 0.2, *p* = 0.633; Time: F = 4.6, *p* = 0.002; Meal × Time: F = 0.2, *p* = 0.945. Controls: Meal: F = 103.7, *p* < 0.001; Time: F = 4.4, *p* = 0.002; Meal × Time: F = 0, *p* = 1. (**D**) Ghrelin (¶, #). T2D: Meal: F = 1.4, *p* = 0.246; Time: F = 11.2, *p* < 0.001; Meal × Time: F = 1, *p* = 0.417. Obese: Meal: F = 2.4, *p* = 0.124; Time: F = 23.5, *p* < 0.001; Meal × Time: F = 1.9, *p* = 0.11. Controls: Meal: F = 1.7, *p* = 0.201; Time: F = 54.3, *p* < 0.001; Meal × Time: F = 0.4, *p* = 0.832

Inflammatory parameters and appetite hormones are presented in Fig. 3. Postprandial secretion of pro-inflammatory TNFα and MCP-1 in T2D and obese participants was not affected by the test meal consumed. However, compared to healthy controls, we observed markedly increased levels of both pro-inflammatory markers in T2D and obese men. In control subjects, V-meal compared to M-meal significantly increased postprandial levels of TNFα (*p* = 0.005), but, compared to T2D and obese men, the levels of TNFα in controls were markedly lower.

The concentrations of leptin were siginificantly higher after the V-meal in T2D (*p* = 0.002), as well as in control subjects (*p *˂ 0.001). Plasma concentrations of leptin were markedly elevated in T2D compared with obese and control subjects, and were the lowest in healthy controls. Plasma concentrations of ghrelin were not significantly different after the test meals in either group and were reduced in T2D and obese men compared to healthy controls. The postprandial dynamics of ghrelin in controls were far more pronounced than in T2D and obese men.

## Discussion

This randomized cross-over study demonstrated that the plant-based V-meal improved postprandial oxidative and dicarbonyl stress markers compared with a conventional energy- and macronutrient-matched M-meal, particularly in men with T2D. We observed significantly decreased levels of GSSG and increased GPx activity in T2D men after the V-meal compared with the M-meal. In obese men, there was an increase in postrandial levels of reduced glutathione and lower postprandial concentrations of methylglyoxal after the V-meal compared with the M-meal. There were no differences in postprandial secretion of TNFα, MCP-1 or ghrelin in T2D or obese men, but we observed a higher postprandial secretion of leptin after the V-meal in T2D men and in healthy controls, compared with the M-meal.

### Oxidative and dicarbonyl stress

The increase in postprandial oxidative and dicarbonyl stress was less pronounced after the V-meal compared with the M-meal. The most prominent differences between both meals occurred in postprandial responses of glutathione and methylglyoxal. The V-meal reduced postprandial amplitudes of oxidative and dicarbonyl stress more than the M-meal. This suggests that a vegan nutrition may offer better protection against the generation of toxic metabolites involved in the development of complications caused by postprandial hyperglycemia and hyperlipidemia. One such toxic metabolite is methylglyoxal. This highly reactive dicarbonyl compound has emerged as a biomarker of diabetes and is closely associated with protein glycation and insulin resistance [18]. The generation of toxic metabolites during hyperglycemia and hyperlipidemia is implicated in the early development of diabetic complications whereby metabolites persist even after glucose and lipid normalization [19]. In our study, we have observed a lower postprandial increase in methylglyoxal after the V-meal in obese individuals, compared with the M-meal. No significant difference between the test meals in terms of methylglyoxal levels was observed in T2D men. However, in T2D patients, postprandial levels of methylglyoxal were markedly elevated compared to obese and control subjects, thus increasing cardiovascular risk. Furthermore, decreased levels of reduced glutathione in T2D patients can induce both oxidative and dicarbonyl stress, while glutathione is involved in methylglyoxal degradation as a co-factor of glyoxalase-1 [7, 8], a glutathione dependent detoxifying enzyme. There is an interplay between the methylglyoxal pathway (its formation and metabolism) and oxidative stress, and glutathione plays an important role in both processes [20]. An increase in methylglyoxal levels seen in T2D men can occur under oxidative stress, probably due to several events including the lower glutathione concentrations. It has been demonstrated that higher plasma concentrations of methylglyoxal are associated with its accumulation in adipose tissue, which may affect the expression and secretion of pro-inflammatory cytokines [21]. These findings are consistent with the observed higher concentrations of methylglyoxal in T2D men in our study.

Bioactive compounds such as polyphenols and other antioxidants might be an important element in the beneficial effects of a plant-based diet on oxidative stress. Dietary polyphenols, a large and heterogeneous group of bioactive compounds, exhibit protective antioxidant properties and also activate transcriptional factor Nrf2. The Nrf2/ARE signalling pathway is an important defence system against exogenous and endogenous oxidative stress injury [22]. In addition to their beneficial dietary effects, polyphenols are understood to possess immunomodulatory, anti-inflammatory [23], and even anti-diabetic properties [24]. Research in this emerging area is ongoing. To the best of our knowledge, a study comparing the post-meal response of methylglyoxal to oxidative stress parameters in T2D patients has not yet been published. Our results in postprandial changes in methylglyoxal and glutathione in obese individuals suggest appropriate targets for an early dietary intervention.

V-meal consumed in our study increased postprandial response in GPx activity in T2D patients, which may help alleviate postprandial oxidative stress. This response was different in T2D men from obese and healthy men. GPx activity can affect glutathione levels and this glutathione-dependent antioxidant enzyme is also involved in the removal of lipoperoxidation products. Although we observed decreased levels of postprandial ascorbic acid in T2D and obese individuals compared with healthy controls, the meal type consumed did not affect the postprandial response in either group (although the ascorbic acid levels tended to be higher after the V-meal compared with the M-meal in healthy controls). Our results are in accordance with previous studies that have shown lower ascorbic acid levels in invividuals who were obese [25], had prediabetes or diabetes [26], suggesting a potential role of adiposity and insulin resistance in lower ascorbic acid levels. Another mechanism that may have affected the lower levels of ascorbic acid in T2D men, is that glucose and ascorbic acid have been shown to compete for entry into the cells; therefore, a postprandial decrease in ascorbic acid would be more associated with hyperglycemia than hyperlipidemia, where glucose inhibits the input of ascorbic acid into the cells [27]. It is also interesting that postprandial levels of ascorbic acid and glutathione had an opposite relationship to each other in dynamics in our study, independent of the test meal.

### Inflammation and appetite hormones

The postprandial state is a condition characterized by low-grade inflammation, whereby cells respond to acute elevations of carbohydrates, triglycerides, and fatty acids [28]. Previous studies have shown that postprandial hyperlipidemia and hyperglycemia increase TNFα levels in healthy and T2D individuals [29], as well as in IGT subjects [30]. In our study, T2D and obese men exhibited increased postprandial levels of both TNF and MCP-1, compared with healthy controls. Therefore, both markers may be sensitive indicators of low-grade inflammation in obese and T2D individuals. However, no differences were observed in MCP-1 following the ingestion of different meals across groups. The slightly increased postprandial secretion of TNFα in the control group following the V-meal may have been due to the higher glucose levels at 60 min after the V-meal compared with the M-meal. However, both glucose and TNFα levels and their postprandial amplitudes in healthy controls were much lower than those in T2D and obese subjects. Due to high fasting and postprandial levels of TNFα in T2D individuals, a ceiling effect could have confounded potential differences between the test meals.

Obese and T2D men in our study exhibited diminished post-meal suppresion of ghrelin secretion and markedly increased postprandial levels of leptin compared with healthy controls. This finding corresponds with previous results reporting postprandial changes in ghrelin to be negatively associated with plasma triglycerides [6]. Plant-based meal stimulated postprandial secretion of leptin more than the conventional meal in T2D and in healthy men. While the action of leptin is essential for energy metabolism, it is also involved in the lipid mobilisation of different fat depots and is understood to protect tissues during lipotoxicity [31]. Lipid oxidation can increase via leptin signalling and has been reported to reduce excess fatty acids [32]. In our study, the increased postprandial response of leptin after the V-meal may have led to such effects in T2D and healthy men, and the lack thereof may have contributed to higher triglyceride levels in obese men after the V-meal. As previously described [18], a plant-based diet increases satiety in T2D patients, and the current findings suggest that postprandial response of the appetite hormones leptin and ghrelin may play an important physiologic role.

### Strengths, limitations, and interpretation

In comparison with the standard oral glucose tolerance test, the two different sandwich meal tests given to the participants in our study contained all the main nutrients, thus increasing physiological stimulation during the post-meal response. This enabled us to identify the mechanism that reflects more than just glucose metabolism. In addition, both meals were served in amounts usually ingested during a typical meal, rendering our results highly applicable. Representing one of the limitations of our study, we did not account for the habitual diets and dietary patterns of the participants when investigating their acute post-meal responses. Also, a longer-term study would be more accurate in determining the merits of a plant-based diet in slowing down and preventing the progression of complications in T2D and obese patients. Nevertheless, we were able to reveal differences in postprandial metabolic mechanisms by comparing single-meal responses, which may assist futher research in preventing the development of associated complications. Also, dietary habits and nutritional status of participants may influence basal levels of some parameters observed during the meal tests.

Oxidative and dicarbonyl stress is a key mechanism in the development of vascular complications during the post-meal phase and should be considered a separate therapeutic target for pharmacological as well as dietary interventions aimed at preventing such complications. A plant-based nutrition may be therefore recommended for T2D and obese individuals.

## Conclusions

In conclusion, our results indicate that the plant-based meal ameliorated the exacerbation of postprandial oxidative and dicarbonyl stress in T2D and obese men compared with a conventional energy- and macronutrient-matched meal, and thus can provide better protection against the development of complications associated with diabetes and obesity. Further studies are needed to verify these effects, in particular after a long-term adherence to a plant-based diet.

## Supplementary Information

**Additional file 1.** Enrollment of the Participants and Completion of the Study.

## Data Availability

The deidentified data will be available upon request at hkahleova@pcrm.org.

## Abbreviations

GPx: Glutathione peroxidase
GSSG: Oxidised form of glutathione
MCP-1: Monocyte chemoattractant protein 1
NEFA: Non-esterified fatty acids
Nrf2: Nuclear factor-erythroid 2-related factor-2
TNFα: Tumour necrosis factor α
