# Supplementary material for: A plant-based meal reduces postprandial oxidative and dicarbonyl stress in men with diabetes or obesity compared with an energy- and macronutrient-matched conventional meal in a randomized crossover study
Source: Nutr Metab (Lond). 2021 Sep 10;18:84. doi: 10.1186/s12986-021-00609-5 (PMC8434736; doi:10.1186/s12986-021-00609-5)
Supplement: Supplementary file 1 — Additional file 1. Enrollment of the Participants and Completion of the Study. [file 12986_2021_609_MOESM1_ESM.pptx]

## Slide 1
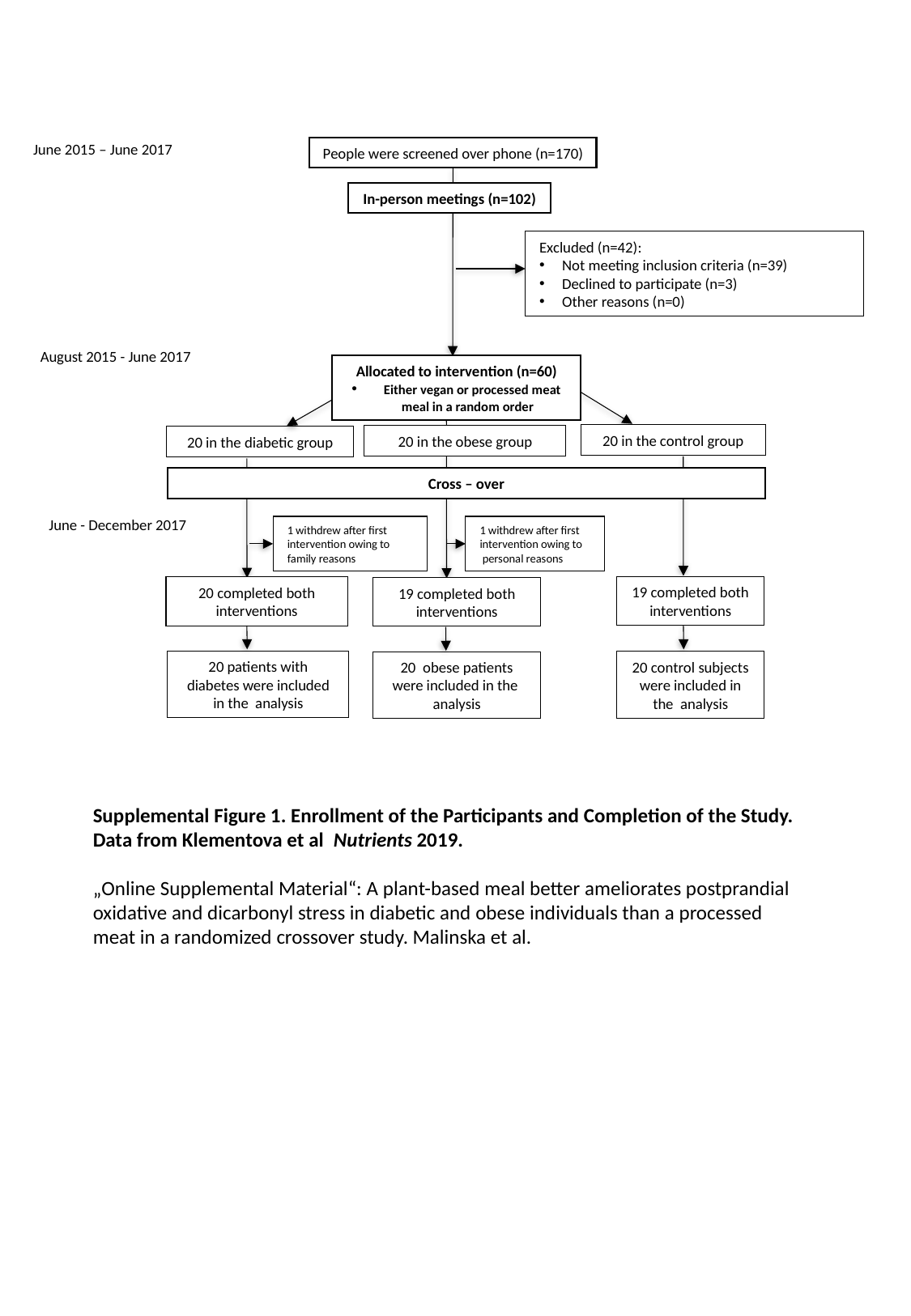

June 2015 – June 2017
People were screened over phone (n=170)
August 2015 - June 2017
20 in the control group
20 in the diabetic group
June - December 2017
1 withdrew after first intervention owing to family reasons
1 withdrew after first intervention owing to
 personal reasons
20 completed both interventions
19 completed both interventions
20 patients with diabetes were included in the analysis
20 obese patients were included in the analysis
In-person meetings (n=102)
Excluded (n=42):
Not meeting inclusion criteria (n=39)
Declined to participate (n=3)
Other reasons (n=0)
Allocated to intervention (n=60)
 Either vegan or processed meat meal in a random order
20 in the obese group
Cross – over
19 completed both interventions
20 control subjects were included in the analysis
Supplemental Figure 1. Enrollment of the Participants and Completion of the Study. Data from Klementova et al Nutrients 2019.
„Online Supplemental Material“: A plant-based meal better ameliorates postprandial oxidative and dicarbonyl stress in diabetic and obese individuals than a processed meat in a randomized crossover study. Malinska et al.
